# High Fill Factors of Si Solar Cells Achieved by Using an Inverse Connection Between MOS and PN Junctions

**DOI:** 10.1186/s11671-016-1678-0

**Published:** 2016-10-10

**Authors:** Liang-Xing Wang, Zhi-Quan Zhou, Tian-Ning Zhang, Xin Chen, Ming Lu

**Affiliations:** 1 Department of Optical Science and Engineering, and Shanghai Ultra-Precision Optical Manufacturing Engineering Center, Fudan University, Shanghai, 200433 China; 2 Thin Film Optoelectronic Technology Center, Shanghai Advanced Research Institute, Chinese Academy of Sciences, Shanghai, 201210 China; 3 Shanghai Institute of Technical Physics, Chinese Academy of Sciences, Shanghai, 200083 China

**Keywords:** Si solar cell, Fill factor, Ag contact, MOS barrier

## Abstract

Fill factors (FFs) of ~0.87 have been obtained for crystalline Si (c-Si) solar cells based on Ag front contacts after rapid thermal annealing. The usual single PN junction model fails to explain the high FF result. A metal/oxide/semiconductor (MOS) junction at the emitter is found to be inversely connected to the PN one, and when its barrier height/*e* is close to the open-circuit voltage of the solar cell, very high FF is obtainable. In this work, although the open-circuit voltage (<580 mV) is not high here, the efficiency of c-Si solar cell still reaches the state-of-the-art value (>20 %) due to the high FF achieved.

## Background

Photoelectric conversion efficiency (*η*) of solar cell is determined by the product of open-circuit voltage (*V*_OC_), short-circuit current density (*J*_SC_), and fill factor (FF); therefore, achieving high *V*_OC_ and *J*_SC_ as well as FF is crucial for getting high *η* [1, 2]. Considering the large abundance of Si on the earth’s crust and the hyper amount of investment input in the Si solar cell industry, new approaches to enhance *V*_OC_, *J*_SC_, and/or FF for further increasing Si solar cell efficiency are always in demand. To our knowledge, FFs for the mostly efficient single crystalline- or c-Si solar cells reported so far are basically no larger than 0.835, for instance, for the PERL-type Si solar cell, FF = 0.828 (*V*_OC_ = 706 mV, *J*_SC_ = 42.7 mA/cm^2^, *η* = 25.0 %) [3], for the IBC-type Si solar cell, FF = 0.830 (*V*_OC_ = 730 mV, *J*_SC_ = 41.2 mA/cm^2^, *η* = 25.0 %) [4], for the HIT-type Si solar cell, FF = 0.835 (*V*_OC_ = 738 mV, *J*_SC_ = 40.8 mA/cm^2^, *η* = 25.1 %) [5], and for the HIT + IBC type Si solar cell, FF *=* 0.827 (*V*_OC_ = 740 mV, *J*_SC_ = 41.8 mA/cm^2^, *η* = 25.6 %) [6]. This work reports our finding of high FF (~0.87) and analyses of its origin based on an inverse connection of metal/oxide /semiconductor (Ag/SiO_2_/Si) or MOS and PN junctions.

## Methods

The substrate of the Si solar cell is P-type c-Si < 100 > wafer (two-sided polish, 10 × 10 × 0.2 mm^3^ in size, 1–5 Ω · cm). First, the Si wafer was degreased and ultrasonically cleaned and then dipped in diluted HF (1 %). Then, it was placed in the boiling NaOH solution with concentration of 1 g/L at 90 °C for 30 min and rinsed in deionized water and blown dry to texture the surface for antireflection. A phosphorous paste was deposited on the front surface of Si, followed by annealing the wafer at temperature of 900 °C for 20 min in a tube furnace in nitrogen with purity of 99.999 % to form PN junction. A 60-nm-thick SiO_2_ was evaporated by means of electron beam heating onto the top surface of now N^+^ Si emitter for surface passivation [7] in a home-made vacuum chamber with a base pressure less than 1 × 10^−4^ Pa. At the rear of the substrate, a 20-nm-thick Al_2_O_3_ was evaporated also by means of electron beam heating for rear passivation [7]. A 1.0-μm-thick Ag grid was then deposited onto the SiO_2_ passivation layer as the front contact by resistance heating in another home-made vacuum chamber with a base pressure less than 5 × 10^−3^ Pa. A 1.0-μm-thick Al layer was deposited onto the Al_2_O_3_ passivation layer as the rear contact by resistance heating in the same system. Finally, the whole device was annealed in nitrogen atmosphere at 510 °C for 2 min followed by rapid thermal annealing (RTA) at 700 °C for 1 s. For RTA, we firstly pushed the sample from one end of the furnace tube to its middle within 3 s. The variation of temperature was from room temperature to 700 °C. Then, the sample was kept in the middle of the tube for 1 s, followed by being pulled out from the middle to the end of the tube within 3 s. All the processes of device fabrication were performed in a clean room. Its degree of cleanness was 100; that means, the number of dust particle with *ϕ* ≥ 0.5 μm was less than 3500/m^3^. The surface morphology was measured on a scanning electron microscope (SEM) (Philips, XL 30). The reflectance spectra were obtained with a spectrophotometer (Perkin-Elemer Lambda 900). The photovoltaic (PV) parameters of solar cells were measured on a solar simulator (Oriel/Newport, model 94023A) under 1-sun AM1.5G condition. In order to confirm the observed results of the high FF, the PV measurements were independently conducted on other two different solar simulators (Oriel/Newport, model 94043A, and Oriel/Newport, model 94023A). The external quantum efficiency (EQE) of the solar cell was acquired on a QE system of Oriel/Newport.

## Results and Discussion

**Fig. 1:**
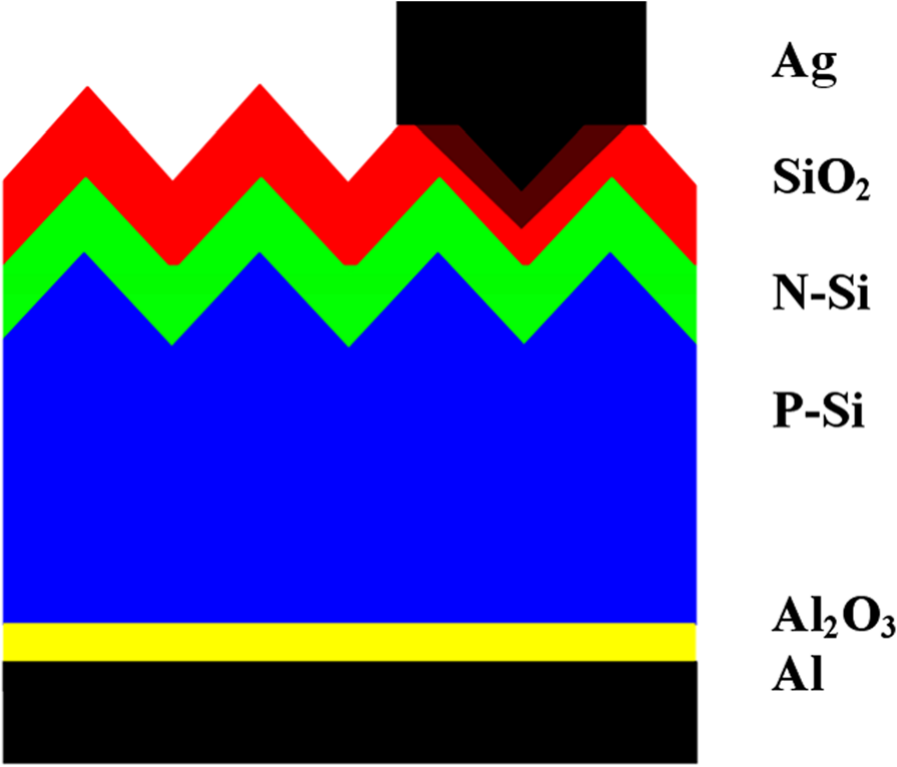
Schematic drawing of c-Si solar cell in this work

**Fig. 2:**
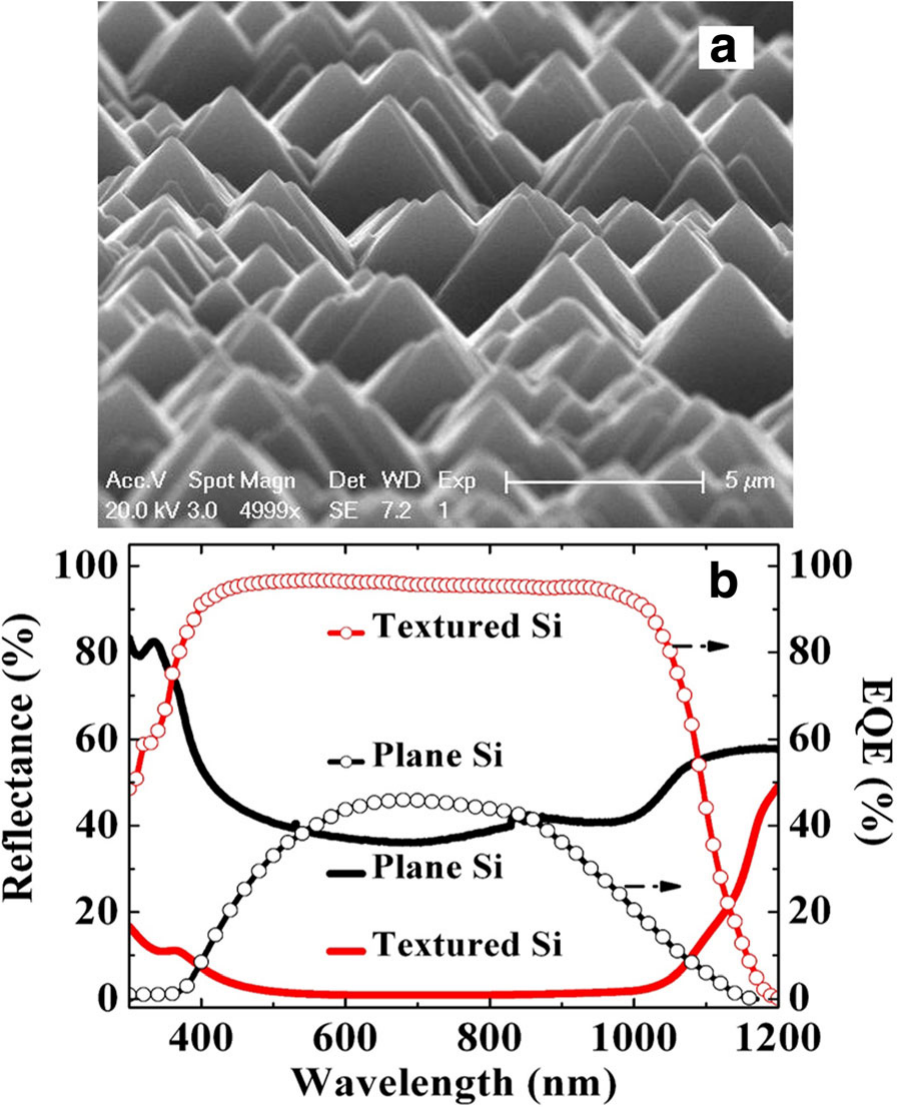
**a** SEM image of Si surface after texturing. **b** Measured external quantum efficiency (EQE) and surface reflectance spectra of Si before and after surface texturing

Figure 1 shows the schematic structure of c-Si solar cell prepared in this work as described above. Figure 2a gives the Si surface image after texturing. The pyramid-like nanostructures with average height of ~2 μm help to trap the incident light [8]. Figure 2b gives the measured EQE spectrum of the textured c-Si solar cell we prepared as shown in Fig. 1, which is termed cell *A*, and that of a plane c-Si solar cell. The surface reflectance spectra of Si before and after surface texturing are also plotted. It is seen that after surface texturing, the reflectance decreases significantly, and the lower reflectance yields higher EQE of the c-Si solar cell.

**Fig. 3:**
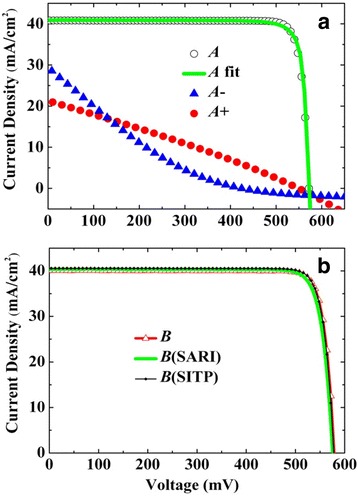
**a** *J-V* curves of cell *A*, those without annealing (*A−*), and over-annealed (*A+*). **b** *J-V* curves of cell *B* measured at Fudan University, those measured at SARI (*B* (SARI)) and SITP (*B* (SITP))

In Fig. 3a, a *J-V* (current density-voltage) curve of c-Si solar cell *A* is shown. Also shown are the *J-V* curves of the same solar cell before the final annealing, which is termed cell *A−*, and that was over-annealed (RTA at 700 °C for 30 s), which is termed cell *A+*. The PV parameters of the three cells are provided in Table 1. For cell *A*, *V*_OC_ = 572 mV, *J*_SC_ = 40.8 mA/cm^2^, FF = 0.869, and *η* = 20.3 %. For cells *A−* and *A+*, the PV parameters all deteriorate severely, especially FF and *η*. Note that the obtained FF (0.869) is considerably larger than the highest experimental one (≤0.835) reported [3–6].

**Table 1:** PV parameters of the c-Si solar cells

Solar cell	*V*_OC_ (mV)	*J*_SC_ (mA/cm^2^)	FF	*η* (%)
*A* (700 °C, 1 s)	572	40.8	0.869	20.3
*A−* (no annealing)	421	29.4	0.187	2.3
*A+* (over-annealing)	553	21.2	0.279	3.3
*B*	576	40.4	0.868	20.2^a^
*B* (SARI)	579	40.1	0.867	20.1^b^
*B* (SITP)	578	40.6	0.867	20.3^c^

^a^Measured at Fudan University

^b^Measured at Shanghai Advanced Research Institute (SARI)

^c^Measured at Shanghai Institute of Technical Physics (SITP)

In order to examine whether a charging effect of capacitance exists during the measurement, the *J-V* curve has been measured by scanning the voltage either from the lower voltage side to the higher one, or from the higher voltage side to the lower one, or to and fro repeatedly. They all remained the same. Hence, no charging effect exists.

Figure 3b gives the *J-V* curves of another solar cell prepared in a similar way as cell *A*, which is termed cell *B*. Its PV parameters are also listed in Table 1, which are quite close to those of cell *A*. Hence, the c-Si solar cell as shown in Fig. 3a is reproducible. Figure 3b also presents the *J-V* curves of the same cell *B* as measured independently on other two different solar simulators in two different affiliations, which are termed cells *B* (SARI) and *B* (SITP), respectively. Here, SARI stands for Shanghai Advanced Research Institute of Chinese Academy of Sciences, and SITP is for Shanghai Institute of Technical Physics of Chinese Academy of Sciences. All these curves are nearly the same. Therefore, the high FF achieved cannot arise from instrumental errors. The PV parameters of the two newly measured *J-V* curves of cell *B* in Fig. 3b are given in Table 1, too.

We now investigate the origin of the very high FF. Firstly the *J-V* curve of solar cell *A* or *B* is analyzed with the usual single PN junction solar cell model as described below [9],

1 $$ J={J}_{\mathrm{ph}}-{J}_0\cdot \left[ \exp \left(\frac{V_{\mathrm{PN}}+J\cdot \mathrm{R}\mathrm{s}}{nVt}\right)-1\right]-\frac{V_{\mathrm{PN}}+J\cdot \mathrm{R}\mathrm{s}}{\mathrm{Rp}} $$

where *V*_PN_ is the voltage across the PN junction. Vt *=* kT*/e*, *T* is absolute temperature, and *e* is electron charge. *J*_0_ means dark current density and *J*_ph_ is photocurrent density. Rs and Rp are serial and parallel resistances, respectively. For the ideal situation when no recombination current exists in the depletion zone, *n,* the ideal factor, equals 1.0 and Rs = 0 and Rp = ∞. In reality, *n* = 1~2.

The experimental *J-V* curve of cell *A* in Fig. 3a is then fitted with Eq. (1). It is found that only when *n =* 0.56 can the calculated curve fit the experimental one well. However, the *n* obtained here (0.56) is far less than 1.0, which is unphysical according to its definition. Recently, Richter et al. [10] have simulated the PV parameters of c-Si solar cell with the model of single PN junction. According to their simulations, it is unlikely to have the very high FF for the relatively low *V*_OC_ as here (<600 mV). Therefore, the model of Eq. (1) is unable to explain the result of very high FF here.

We notice that the c-Si solar cell is actually composed of a PN junction and two metal/oxide/semiconductor or MOS junctions (Ag/SiO_2_/N^+^ Si and Al/Al_2_O_3_/P-Si). If the barrier heights of the two MOS junctions are very low, or if they are in Ohmic contacts with the PN junction, the *J-V* curves of Si solar cell can still be described by Eq. (1) [11]. This is surely not the case according to the analysis above. In fact, the Ag/SiO_2_/N-Si junction is inversely connected to the PN one; that is, the internally built-in fields of the two junctions are reverse in direction; while, Al/Al_2_O_3_/P-Si junction would be either forwardly connected to the PN one; that is, the built-in fields of the two junctions are the same in direction or be in Ohmic contact with the PN junction considering the P-type feature of the substrate [9–11]. The *J-V* dependence of MOS junction cell can be described by Eq. (2) in the following [9, 11–17],

2 $$ J={J}_s\cdot \exp \left(\frac{V_{\mathrm{MOS}}}{\mathrm{ns}\cdot \mathrm{V}\mathrm{t}}\right)\cdot \left[1- \exp \left(-\frac{V_{\mathrm{MOS}}}{\mathrm{Vt}}\right)\right] $$

where*J*_*s*_ = A* ⋅ *T* ^2^ ⋅ exp(−*φ*_*B*_/Vt). *A* ^*^ means effective Richardson constant. *ϕ*_*B*_ is the barrier height. ns is ideal factor of the MOS junction. For N-Si, *A* ^*^ = 112 Acm^−2^K^−2^, and for P-Si, *A* ^*^ = 32 Acm^−2^K^−2^ [17].

**Fig. 4:**
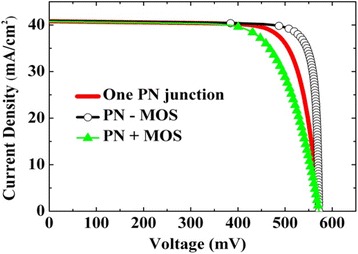
Simulated *J-V* curves of one PN junction solar cell. PN junction forwardly connected to MOS (PN + MOS) and PN junction inversely connected to MOS (PN-MOS)

Figure 4 depicts the simulated *J-V* curve of one PN junction solar cell, as calculated with Eq. (1) by using PV parameters of *V*_OC_ = 572 mV, *J*_SC_ = 40.8 mA/cm^2^, *n* = 1.06, Rs = 0.11 Ω · cm^2^, and Rp = 8552 Ω · cm^2^. The PV parameters selected are taken from the fitting results as will be explained in the following. The *J-V* curve of the MOS junction solar cell was calculated with Eq. (2) by using the same *J*_SC_ (40.8 mA/cm^2^), with *ns* = 1.21, and *ϕ*_*B*_ = 0.505 eV, which are also from the following fitting results. For the case of forward connection, or the model of PN + MOS,

3 $$ V={V}_{\mathrm{PN}}+{V}_{\mathrm{MOS}} $$

and for the case of inverse connection, or the model of PN-MOS,

4 $$ V={V}_{\mathrm{PN}}-{V}_{\mathrm{MOS}} $$

To simulate the *J-V* curve of PN + MOS or PN-MOS, a numerical calculation procedure was used; that is, starting from *J* = *J*_SC_ down to *J* = 0, for each point of *J* (the step of *J* was chosen 0.1 mA/cm^2^), both *V*_PN_ and *V*_MOS_ were calculated by Eqs. (1) and (2), respectively, combining all the other selected PV parameters. Then, Eqs. (3) and (4) were applied to get the total voltage across the solar cell for the models of forward and inverse connections, respectively. The resulting *J-V* curves of PN + MOS and PN-MOS are given in Fig. 4, too. It is clear that for the forward connection, FF even becomes lower; while, for the inverse connection, higher FF is available. It should be pointed out that it is only when *ϕ*_*B*_/*e* of the MOS junction is not far from *V*_OC_ of the solar cell, which is the case during the simulation of Fig. 4, could a high FF be obtained. In fact, we have simulated the relation between FF and *ϕ*_*B*_. It is found that when *ϕ*_*B*_/*e* is very close to or even equals *V*_OC_, FF larger than 0.9 can be obtained.

We then use the model of PN-MOS to fit the *J-V* curve of cell *A* with Eqs. (1) and (2). The fitting curve has been plotted in Fig. 3a. At this moment, the derived PV parameters are *n* = 1.06, ns = 1.21, *ϕ*_*B*_ = 0.505 eV, Rs = 0.11 Ω · cm^2^, and Rp = 8552 Ω · cm^2^. The values of *n* and ns are now rational, and *ϕ*_*B*_/*e* is close to *V*_OC_ (although not very close to 0.572 V), which is consistent with the inference derived above.

It needs to be pointed out that all the PV parameters including FF are very sensitive to the annealing conditions, as have been illustrated in Fig. 3a. If there is no annealing or the annealing is insufficient, the barrier height and the serial resistance would become larger [18–20], which could severely degrade the performance of solar cell. On the other hand, if the annealing is overdone, Ag will easily penetrate through SiO_2_ and into the emitter zone or even into the PN junction, then the whole device would be ruined. The RTA is mainly used to modulate the barrier height of MOS junction, so a proper RTA is crucial to get a very high FF. On the other hand, the thickness of SiO_2_ also influences FF as well as *V*_OC_ and *J*_SC_, via influencing Ag diffusion and barrier height of the MOS junction. An optimized combination of annealing and SiO_2_ thickness has been performed here to achieve a high efficiency.

## Conclusions

In summary, we have obtained high FFs (~0.87) of c-Si solar cells by using Ag contact as front electrode after proper annealing and rapid thermal annealing treatments. Our model analysis indicates that the very high FF is caused by the inverse connection of MOS (Ag/SiO_2_/Si) and the PN junctions, with the MOS junction possessing a barrier height/*e* close to *V*_OC_. By using this approach, c-Si solar cells with efficiencies >20 % are achieved due to their high FFs, although their open-circuit voltages are not high (<580 mV) here. It is expected that further improvements on increasing open-circuit voltage and meanwhile remaining the very high FF could lead to further higher efficiency.

## Abbreviations

*e*: Electron charge
EQE: External quantum efficiency
FFs: Fill factors
*J*_0_: Dark current density
*J*_ph_: Photocurrent density
*J*_SC_: Short-circuit current density
MOS: Metal/oxide/semiconductor
*n*: The ideal factor of the PN junction
ns: Ideal factor of the MOS junction
PV: Photovoltaic
Rp: Parallel resistance
Rs: Serial resistances
RTA: Rapid thermal annealing
SARI: Shanghai Advanced Research Institute
SEM: Scanning electron microscope
SITP: Shanghai Institute of Technical Physics
*T*: Absolute temperature
*V*_OC_: Open-circuit voltage
*η*: Conversion efficiency
*ϕ*_*B*_: The barrier height
